# A quantitative detection of Cardiotrophin‐1 in chronic heart failure by chemiluminescence immunoassay

**DOI:** 10.1002/jcla.23570

**Published:** 2021-03-13

**Authors:** Ying Ping, Xuchu Wang, Yibei Dai, Danhua Wang, Weiwei Liu, Pan Yu, Zhihua Tao

**Affiliations:** ^1^ Department of Laboratory Medicine The Second Affiliated Hospital of Zhejiang University School of Medicine Hangzhou China

**Keywords:** Cardiotrophin‐1, chemiluminescence immunoassay, chronic heart failure, paramagnetic particles, quantitative detection

## Abstract

**Background:**

Cardiotrophin‐1 (CT‐1) is a cytokine that could induce cardiomyocytes hypertrophy and dysfunction. Plasma CT‐1 might serve as a cardiac biomarker both in diagnosis, staging, and prognostic assessment of heart failure.

**Methods:**

In this study, a one‐step paramagnetic particles‐based chemiluminescence immunoassay (MPs‐CILA) for rapid and sensitive detection of plasma CT‐1 was established. Plasma samples were directly incubated with biotin‐labeled anti‐CT‐1 antibody (bio‐Ab) and acridine ester labeled anti‐CT‐1 antibody (AE‐Ab) to form sandwiched complex. The sandwiched CT‐1 was then captured by streptavidin modified paramagnetic particles (MPs‐SA) for rapid separation and signal generation.

**Results:**

The proposed MPs‐CLIA presents a laudable linear relationship ranging from 7.8 pg/mL to 200 ng/mL with a detection limit of 1.0 pg/mL. The recoveries of spiked human plasma samples at low (10pg/mL), medium (100 pg/mL), and high (800 pg/mL) levels of CT‐1 were 96%, 104%, and 110% respectively. The intra‐analysis coefficient variation (CVs) of the 3 samples was 8.92%, 6.69%, and 3.54%, respectively. And the inter‐analysis coefficient variation (CVs) was 9.25%, 10.9%, and 4.3%, respectively. These results strongly indicate high sensitivity, wide linear range, acceptable precision, and applicable reproducibility of the proposed method to detect plasma level of CT‐1. Finally, Plasma CT‐1 from 140 subjects with or without chronic heart failure was analyzed to assess the clinical application of MPs‐CILA.

**Conclusions:**

Noteworthily, the MPs‐CLIA method is highly automated such that it is suitable for high‐throughput detection of CT‐1 in clinical inspection.

## Graphical abstract

A one‐step paramagnetic particles‐based chemiluminescence immunoassay (MPs‐CILA) for rapid and sensitive detection of plasma CT‐1 was established, and plasma CT‐1 from 140 subjects with or without chronic heart failure was analyzed to assess the clinical application of MPs‐CILA.

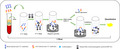

## 1 INTRODUCTION

Chronic heart failure (CHF) is a global public health concern greatly impacting patient's quality of life, and inflicting significant morbidity and mortality.^1^ As CHF is often characterized by obscure symptoms, associating with a variety of pathophysiological and biochemical diseases, its early diagnosis is challenging.^2^, ^3^ BNP or pro‐BNP has identified as an objective indicator for the diagnosis of heart failure and is currently recommended by the European Society of Cardiology (ESC) guidelines for CHF diagnosis.^4^ However, BNP/pro‐BNP significantly varies regarding non‐cardiac factors, such as age, gender, race, renal function result in low specificity for CHF diagnosis.^5^, ^6^, ^7^, ^8^, ^9^ Cardiotrophin‐1 (CT‐1) is a member of interleukin‐6 superfamily with molecular mass of 21.5KD.^10^ CT‐1 protein is abundantly expressed in heart tissue, its overexpression is mainly stimulated by ventricular stretch/pressure,^11^ which promotes cardiac hypertrophy^12^ and myocardial fibrosis ^13^, ^14^via binding to gp130/LIF complex,^15^, ^16^ eventually participates the progression of chronic heart failure.^13^ It is predominantly secreted by myocardium cell through coronary sinus into the peripheral circulation.^17^ A plethora of studies present that CT‐1 level in heart and plasma were significantly elevated in CHF patients.^18^, ^19^, ^20^ Tsutamoto et al ^21^ have demonstrated that plasma levels of CT‐1 increased with the severity of CHF, and high plasma levels of CT‐1 associated with high mortality in CHF patients. Moreover, compared with BNP/pro‐BNP, CT‐1 is independent of age, gender, BMI, and renal function.^18^ Considering the importance of CT‐1 to the presence and severity of CHF, developing an automated detection platform for accurately and rapidly quantifying CT‐1 at low concentration possesses a great value.

Thus far, various analytical methods have been reported to be used detect CT‐1. In 1999, Talwar et al ^22^ developed a competitive immunoluminometric assay, firstly quantitative assessment of plasma CT‐1 in humans. The method requires a pre‐analysis step as long as 24 hours, and due to the extra extraction step, its specificity is lower and it is more prone to errors. Some methods proposed later were abandoned due to complicated operation process, long detection period, and high detection cost.^17^, ^21^, ^23^

Currently, chemiluminescence immunoassay is increasingly used in ultramicro‐analysis of biological substances due to the advantages of being extreme sensitivity, high specificity, good reproductivity, and simplicity, which has basically replaced radioactive immunoassay and enzyme immunoassay technology.^24^ In this study, we constructed a paramagnetic particles‐based chemiluminescence immunoassay (MPs‐CLIA) for rapid determination of cardiotrophin‐1 in plasma. We adopt streptavidin‐coated paramagnetic particles (MPs‐SA) in combination with the capture antibody (biotin‐labeled anti‐CT‐1 antibody, bio‐Ab) to separate the immune complexes from the complex matrix, which considerably improves the efficiency of the separation and washing steps, and provides a simplified procedure.^25^, ^26^, ^27^ After separation by magnetic separation column, the immune‐complexes were recovered for the quantitative CT‐1. In the immunoassay scheme, chemiluminescence signal produced by an anti‐human free CT‐1 antibody labeled with acridinium ester (AE‐Ab) was directly proportional to the amount of CT‐1 in a sample. The chemiluminescent acridinium ester labels have excellent chemiluminescent properties such as low background signal, high detection sensitivity and no need for a catalyst and thus efficiently simplify the detection procedure.^28^

## 2 MATERIAL AND METHODS

### 2.1 Apparatus and reagents

A photomultiplier instrument for chemiluminescence signal detection was developed by our laboratory. Electrochemiluminescent immunoassay instrument (ROCHE/Cobas e 601) was provided by Clinical Laboratory of Zhejiang university second affiliated hospital. The incubation procedures were carried out using a constant temperature incubator. A magnetic separation device was purchased by Thermo Fisher Scientific (Invitrogen).

Human recombinant cardiotrophin‐1 (hrCT‐1) in its pure form used in the study was purchased from Sino biological (Sino Biological Inc). Rabbit anti‐human CT‐1 polyclone antibody pair (db1927) including detector and capture antibody were provided by Hangzhou Diag Biotechnology Co., Ltd. (China). Streptavidin‐modified paramagnetic particles were purchased from Thermo Fisher Scientific (Invitrogen). N‐hydroxysuccinimide biotin, acridinium ester, bovine serum albumin (BSA), phosphate buffer saline (PBS), Tween‐20, dimethylsulfoxide (DMSO), and anhydrous dimethyl formamide (DMF) were purchased from Sigma‐Aldrich Ltd (USA). Hydrogen peroxide/sodium hydroxide reagent was prepared by ourselves. Washing buffer was made by PBS containing 0.1% Tween‐20 and 0.1% BSA. All Double distilled water was prepared using water purified with an ultrapure water system (Zhejiang university second affiliated hospital). All of the other chemicals were standard commercial products of analytical‐reagent grade.

### 2.2 Patient recruitment

To verify the clinical applicability of our method, 100 patients hospitalized with chronic CHF (72 men and 28 women) aged between 22 and 84 years (mean 60) were recruited at our institutions and quantified CT‐1 from these samples by MPs‐CLIA (Table 1). The plasma pro‐BNP concentrations from 44 plasma samples were measured with commercially electrochemiluminescent immunoassay kit (https://dialog1.roche.com/cn/zh_cn/elabdoc). The cause of heart failure was dilated cardiomyopathy in 49 patients, ischemic heart disease in 36 patients, valvular heart disease in 11 patients, hypertensive heart disease in 2 patients, and hypertrophic cardiomyopathy in 2 patients. All patients had a left ventricular ejection fraction below 50% on color doppler echocardiography. Patients with acute myocardial infarction, infection, chronic inflammatory disease, malignancy, or renal failure were excluded. All patients were clinically stable on constant doses of diuretics, 41 patients were treated with β‐blockers. A group of 40 healthy subjects with the same age and sex distribution of the study group recruited in the Second Affiliated Hospital of Zhejiang University School of Medicine. Research protocols were approved by the Ethical Committees for Clinical Research of supporting institutions, and all patients provided written informed consent for the use of their samples for experimentation.

**Table 1:** Clinical and echocardiographic characteristics of the study group

Variables	HF patients	Healthy subjects
Age(years)	22‐84	66 ± 13
Sex(male/female)	72/28	27/13
BMI (kg/m^2^)	18.9 ± 3.4	19.5 ± 3
HF (beats/min)	74 ± 5	72 ± 5
Systolic pressure	118 ± 23	116 ± 30
Diastolic pressure	71 ± 17	72 ± 10
LVEF (%)	<50%	>70%
Treatments		
β blockers, n, (%)	41(41%)	
Diuretic, n, (%)	100(100%)	

Note

Data are presented as mean ± standard deviation or as absolute numbers (n) with percentage (% of the referring group) in brackets.

Abbreviations: BMI, Body Mass Index; HF, heart rate; LVEF, left ventricular ejection fraction.

Collected plasma using EDTA or heparin as an anticoagulant, plasma was centrifuged for 10 minutes at 3000 × g, store samples in aliquot at −80°C for later use. Avoid repeated freeze/thaw cycles.

### 2.3 Preparation of Biotin‐labeled anti‐human free CT‐1 antibody (bio‐Ab)

Preparation of bio‐Ab was conducted through the reaction between N‐hydroxysuccinimide biotin and anti‐human free CT‐1 antibody. As described previously with a few changes,^29^ firstly, the antibody solution was diluted with half‐volume of 0.1 M sodium tetraborate/0.1 M NaCl (PH: 8.5). The N‐hydroxysuccinimide biotin was dissolved in DMSO (2.8 mg/L). Thereafter, the antibodies solution and excess of the N‐hydroxysuccinimide biotin with either 5:1, 10:1, or 25:1 molar were mixed with magnetically stirring at room temperature for 1 hour. Finally, the biotinylated antibodies (bio‐Abs) were separated from the free biotin using sephadex G‐75 chromatography column. The purified bio‐Abs solution containing 0.02% sodium azide and 0.25% BSA was stored at −20°C.

### 2.4 Preparation of acridinium ester labeled anti‐human free CT‐1 antibody (AE‐Ab)

Briefly, anti‐human free CT‐1 antibody solution was diluted to 0.16 mg/mL by 0.1 M PBS (PH:7.4). Then, the antibody solution was reacted with 10 μL acridinium ester solution (0.5mM) previously dissolved in anhydrous dimethyl formamide (DMF). The reaction solution was mixed well at room temperature for 30min. The AE‐Ab solution was also purified by sephadex G‐75 Chromatography column. The purified AE‐Abs solution containing 0.02% sodium azide and 0.25% BSA was stored at −20℃.

### 2.5 MPs‐CLIA procedure

**Figure 1:**
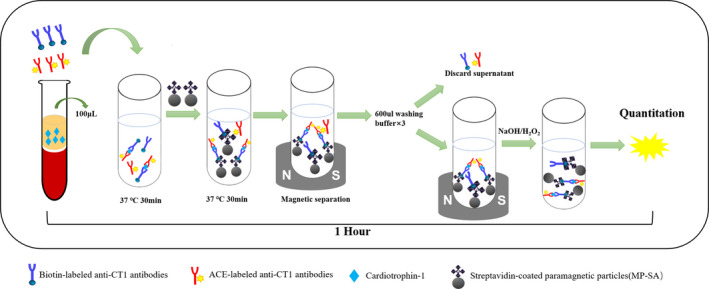
Schematic illustration of the quantitative detection of CT‐1 by MPs‐CLIA. 50 μL bio‐Ab (capture antibody) (1.6 μg/ mL), 50 μL AE ‐Ab (detector antibody) (1.6 μg/mL) was incubated with 100 μL CT‐1 in PBS or plasma at 37°C for 30 min. Thereafter, MPs‐SA was incubated with bio‐Ab‐CT‐1‐AE‐Ab sandwich immunocomplexes and then unreacted antibodies were removed by magnetic separation. After washing three times, The luminescence signal was generated by the trigger solution containing 200 μL acid buffer with oxidants hydrogen peroxide (H_2_O_2_) and 200 μL sodium hydroxide (NaOH). Then the relative light unit (RLU) was measured with a photomultiplier instrument to quantify CT‐1 concentration

MPs‐CLIA was based on double antibodies, which specifically recognizes CT‐1. As is shown in Figure 1, a “one‐step” reaction, 50 μL bio‐Ab (capture antibody) (1.6 μg/ mL) in 5% BSA‐PBS, 50 μL AE ‐Ab (detector antibody) (1.6 μg/mL) in 5% BSA‐PBS, and 100 μL CT‐1 in PBS or plasma were stepwise pipetted into the reaction tube, followed by incubation at 37°C for 30 minutes. Then bio‐Ab‐CT‐1‐AE‐Ab sandwich immunocomplexes are formed, thereafter MPs‐SA was added into each well to facilitate the magnetic separation. After another 30 minutes of incubation at 37℃, the cuvette was then placed on a magnetic plate for 1 minutes, the sandwich immunocomplexes bound to MPs‐SA was magnetically separated. Next, the supernatant was discarded, washing buffer (600 μL) was added to the reaction cuvette and repeatedly washed three times. Without any further steps after extensive washing, the trigger solution containing 200 μL acid buffer with oxidants Hydrogen peroxide(H_2_O_2_) and 200 μL sodium hydroxide (NaOH) was added to the well stepwisely for luminescence signal generation. Then the relative light unit (RLU) was measured with a photomultiplier instrument to quantify CT‐1 concentration. **Figure** **1** shows a schematic diagram of the experimental procedure.

### 2.6 Statistical analysis

Continuous variables are presented as mean ± standard deviation (SD) or median ± inter‐quartile range (IQR), and significant differences were determined with Mann‐Whitney test. Levels of statistical significance were set at *P* < .05 (*) as indicated. All the data were analyzed by SPSS Statistics 20.0 and GraphPad Prism 8. Furthermore, CT‐1 concentrations in individuals with heart failure vs those in normal individuals were compared by use of Scatter plot analysis. At least double replicates for each CT‐1 concentrations were performed.

## 3 RESULTS AND DISCUSSION

### 3.1 Optimization of MPs‐CLIA experimental condition

**Figure 2:**
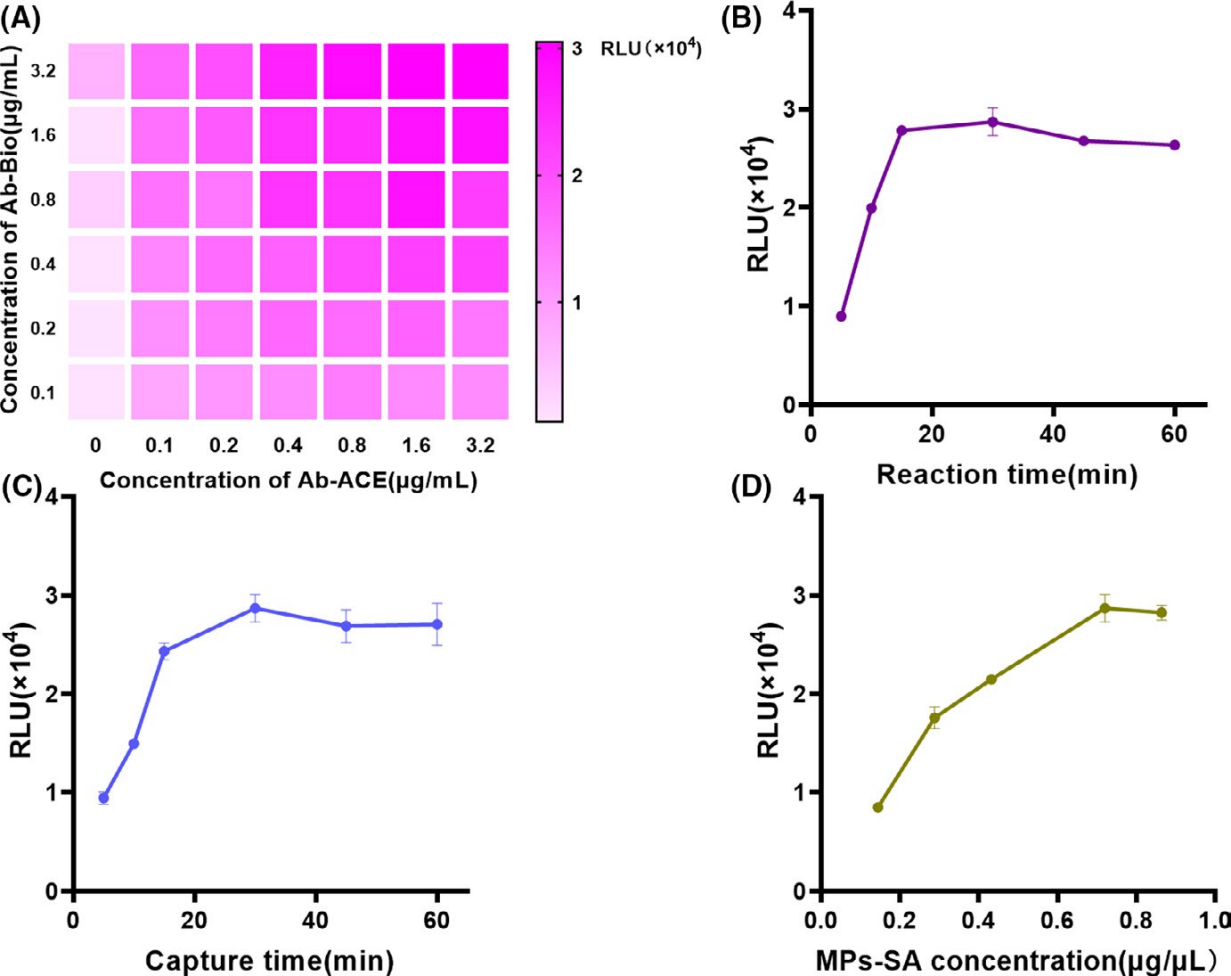
Optimization of crucial parameters for CT‐1 detection by MPs‐CLIA. A, antibody concentrations. Color represents relative light unit, the darker the color, the higher the relative light unit. B, incubation time, closed circles represent the incubation times of 5, 10, 15, 30, 45 min, and 1 h; C, capture time, closed circles represent the incubation times of 5, 10, 15, 30, 45 min, and 1 h; D, magnetic particle concentration, closed circles represent streptavidin‐modified paramagnetic particles concentrations of 0.14, 0.28, 0.43, 0.72, and 0.86 μg/μL

For the sake of the superior performance of MPs‐CLIA, a series of reaction parameters were studied and optimized in MPs‐CLIA, including antibody concentrations, time for incubation, MPs‐SA concentration. The concentration of the antibody pair was the key factors affecting the sensitivity and specificity of the immunoassay. We first optimized the assay concentration of antibody pair, the purified CT‐1 analyte (1000 pg/mL) was coupled to a range of concentrations of antibody pair at 0.1,0.2, 0.4, 0.8, 1.6, and 3.2 μg/mL, the combination yielding the greatest signal‐to‐noise ratio was selected. Figure 2A shows the luminescence value increased with the increasing of double anti‐CT‐1 antibodies concentration and reached a maximum when the concentration was 1.6 μg/mL. Thus, 1.6 μg/mL was adopted as the optimal concentration of double antibodies. It is well known that incubation time is also an important factor in the reaction of antigens and antibodies. The optimal of antigen‐antibody immune reaction time and paramagnetic particles capture time were determined by MPs‐CLIA, in which various reaction time were examined. Figure 2B exhibits the RLU changes in different reaction times at 5, 10, 15, 30, 45, and 60 minutes. With immunoreaction time increased, the RLU increased up to 15 minutes, and then leveling off from 15 to 60 minutes. Thus 15 minutes was selected as the optimal condition for MPs‐CLIA downstream experiments. As the same, Figure 2C suggests that the occurrence of reaction equilibrium between MPs‐SA and immune complex was in the range of 30‐60 minutes, so 30 minutes was chosen as the best capture time. The systematic investigation of the optimal conditions revealed that the present MPs‐CLIA can be performed within ~1 hour, this operation time is 4 times more rapid than conventional ELISA (~4.5 hours). The concentration of MPs‐SA was investigated by MPs‐CLIA using an excess amount of CT‐1 and various concentrations of MPs‐SA (0.14, 0.28, 0.43, 0.72, 0.86 μg/μL). As is shown in Figure 2D, the RLU increased along with the concentration of MPs‐SA and reached a plateau at 0.72 μg/μL (50 μL), indicating that the amount of MPs‐SA was saturated. Hence, the following experiment was executed with MPs concentration of 0.72 μg/μL.

After optimizations, the conditions of the methodology have been determined, the following experiments were carried out according to the above scheme.

### 3.2 Methodology evaluation

#### 3.2.1 Linear curve

**Figure 3:**
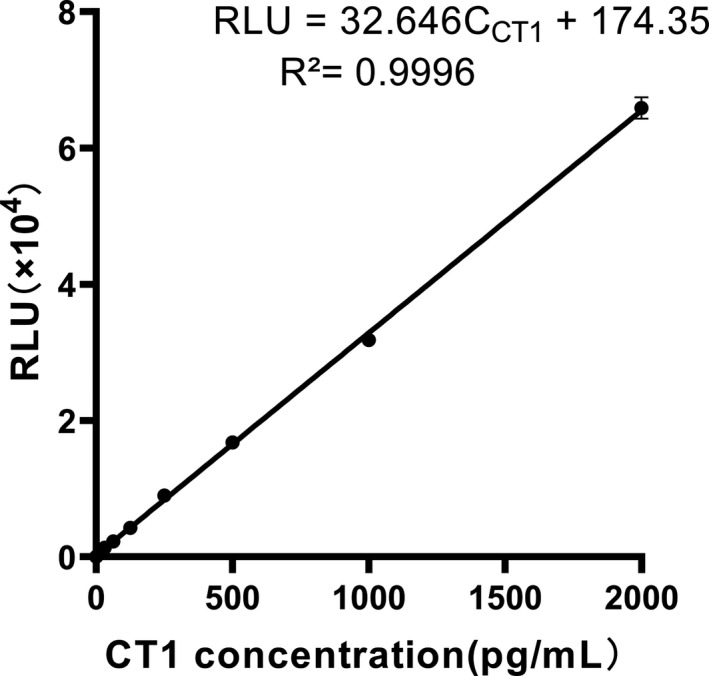
Calibration curve for the determination of CT‐1 by MPs‐CLIA. MPs‐CLIA was conducted using bio‐Ab and AE‐Ab at concentration of 1.6 μg/mL, respectively. Reaction time and capture time were 15 and 30 min, respectively. The dotted line represents the linearized curve ranging from 7.8 to 2000 pg/mL. RLU: relative light unit; C_CT‐1_: the concentration of CT‐1

Recombinant human CT‐1 in the form of freeze‐dried powder was dissolved with sterile PBS conducted by the manufacturer protocol. The serially diluted calibration samples in human plasma matrix (7.8, 15.6, 31.25, 62.5, 125, 500, 1000 pg/mL) were measured using MPs‐CLIA. Under the optimal conditions of exploration, standard curve obtained was $\mathrm{RLU}=32.646C_{CT1}+174.35$ with linear range of 7.8pg/mL‐200ng/mL and a good determination coefficient of 0.9996 (Figure 3). This range of concentrations quantification is far greater than the pathological level of CT‐1, which enough provides clinically useful and sensitive information without pre‐dilution of specimens.

#### 3.2.2 The limit of detection

The minimum detection limit is among the most important characteristics of applying a method to a clinical sample and obtaining a minimum number of false negatives. The calculated limit of detection (LOD) was determined from assay of 10 replicates of the zero calibrator with three times and calculated from the experimental result. LOD can be expressed as *LOD = 3Sa/b* where Sa is the standard deviation of the response and b is the slope of the calibration curve.^30^ The detection limit was calculated as 1.0 pg/mL. Several immunoassay methods for plasma level of CT‐1 were summarized in Table 2. Compared with the previous reports, MPs‐CLIA presents relatively wider linear range, a lower detection limits and shorter detection time.

**Table 2:** Comparison between MPs‐CLIA and the previous reported detection methods

Methods	Time (hour)	LOD	linear range	reference
ELISA	~4	5.6pg/mL	16‐2000pg/mL	^13^
ILAM	>24	1.1 ± 0.8fmol/mL	N.F	^22^
RIA	>16	N.F	120‐8300fmol/mL	^17^
CIA	>24	2.9fmol/mL	N.F	^23^
MPs‐CLIA	~1	1pg/ mL	7.8‐2000pg/mL	Present study

Abbreviations: CIA, Non‐competitive immunochemiluminometric assay; ELISA, Enzyme‐linked immunosorbent assay; ILAM, Competitive immunoluminometric assay; N.F, No reference; RIA, Competitive radioimmunoassay.

#### 3.2.3 Analysis of the accuracy

To evaluate the accuracy of MPs‐CLIA, recovery experiment was performed by spiking various amounts of CT‐1 (10 pg/mL, 100 pg/mL, 800 pg/mL) into pooled plasma samples and analyzed immediately. Simultaneously, eight concentrations within the range of linearity (1000, 500, 250, 125, 62.5, 31.25 16.5, and 7.8 pg/mL) were analyzed, obtaining a calibration curve of the day. The spiked amount of CT‐1 is determined through MPs‐CLIA. The RLU of spiked samples was interpolated in the calibration curve obtained for the day and calculated the detected value. The recovery rate (RA) was derived from the measured and spiked amounts of CT‐1. The RA was calculated as follows: recovery rate (%) =100 × spiked amounts/ detected values. As is shown in Table 3, the results showed that the CT‐1 at different levels were recovered within 96%, 104%, 110%, suggesting that the developed MPs‐CLIA can apply for the accurate and reliable determination of CT‐1 in plasma samples without the interferences of other components in human plasma samples.

**Table 3:** Results of CT‐1 determination in spiked serum/plasma samples Calibration equation: $\mathrm{RLU}={32.646C}_{CT1}+174.35$ $R^{2}$= 0.9996 Results are the mean of n = 10 measurements

Spiked CT1 concentration (pg/mL)	Calculated CT1 concentration (pg/mL)	Mean recovery (%)	RSD (%)
10	9.6	96%	8.1
100	104	104	6.3
800	885	110	4.2

#### 3.2.4 Precision

The precision of the MP‐CLIA was estimated based on repeated measurement of healthy human pooled plasma spiked with calibrator CT‐1. Three different concentrations of CT‐1 calibrator (10, 100, and 800 pg/mL) were prepared under the same conditions. The intra‐analysis precision was calculated by analyzing each concentration 10 times per run in one day (n = 10). Similarly, these samples were analyzed 10 times for three days (n = 30) to obtain the inter‐analysis. As shown in Table 4, the intra‐analysis CVs of the three samples enrolled were 8.92%, 6.69%, and 3.54%, respectively, and inter‐analysis CVs were 9.25%, 10.9%, and 4.3%, respectively. The CVs are acceptable for the very low concentration of CT‐1 measured, at the pg/mL order. Such reproducibility is highly acceptable and in favor of the MPs‐CLIA assay.

**Table 4:** Intra‐assay and inter‐assay of MPs‐CLIA

CT‐1 concentration (pg/mL)	CVs (%)	
	inter‐assay (n = 30)	intra‐assay (n = 10)
10	9.25%	8.92%
100	10.9%	6.69%
800	4.30%	3.54%

Note

Coefficient of variation (CV)=100 × mean/standard deviation.

#### 3.2.5 Plasma CT‐1 detection in clinical sample

**Figure 4:**
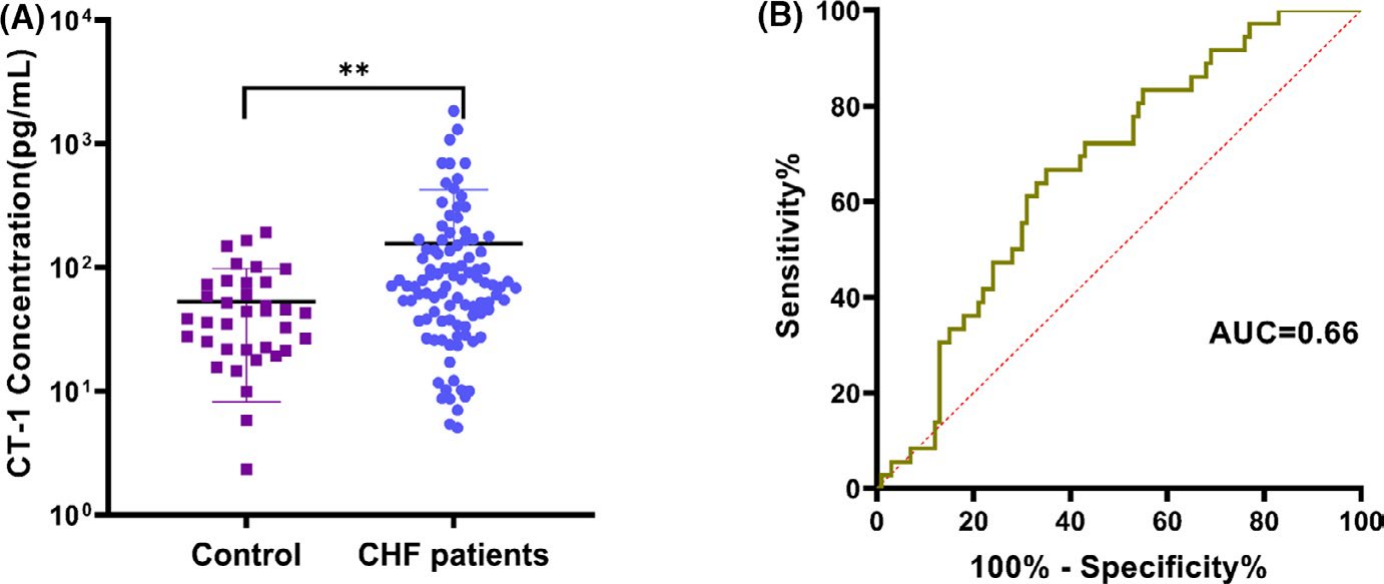
CT‐1 plasma level in 100 chronic heart failure patients and 40 controls. A, Comparison between healthy people and CHF patients, (*P* < .05). B, Receiver operating characteristic curves for the ability of cardiotrophin‐1 (CT‐1) to diagnosis in CHF patients. The area under the ROC curve gave a value of 0.66. A threshold value of 52.06 pg/ mL gives a sensitivity of 63.5% and a specificity of 65%

**Figure 5:**
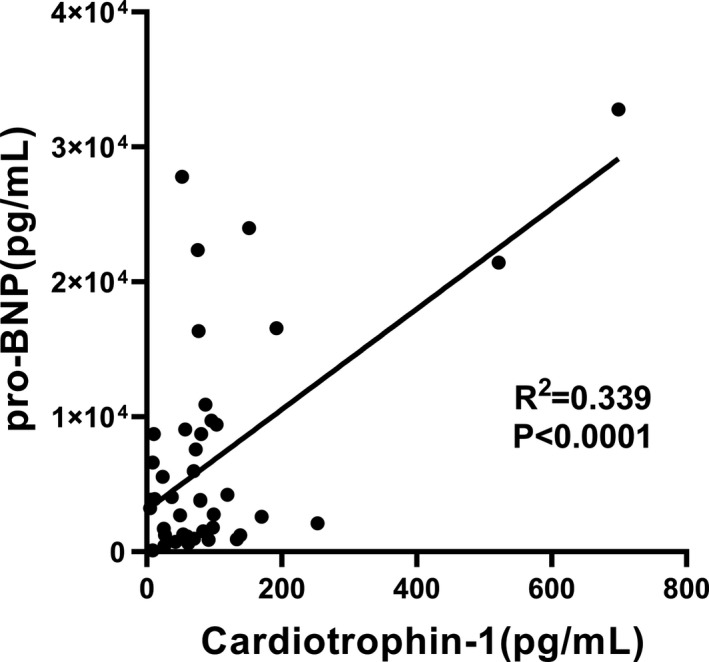
Correlation between plasma levels of CT‐1 and pro‐BNP in 44 CHF patients

Plasma CT‐1 levels from 100 CHF patients and 40 subjects were determined to validate the clinical application of MP‐CLIA. It shows that the CT‐1 concentrations were significantly higher in CHF patients (median: 70.43pg/mL) compared with healthy individuals (median: 40.70 pg/mL) (*P* < .05, Figure 4A). ROC curve was further performed to evaluate the diagnostic value of CT‐1, which yielded an area under the curve of 0.66 (Figure 4B). In the cutoff of 52.06pg/mL, the sensitivity and specificity are calculated as 63.5% and 65%, respectively. The relationship between levels of plasma CT‐1 and pro‐BNP was studied. As is shown in Figure 5, regression analysis revealed significant association between CT‐1 and pro‐BNP (*r* = 0.339, *P* < .001). In addition, we observed that 3 CHF patients who eventually died maintain far higher CT‐1 levels. In this study, we found that CT‐1 is a potential auxiliary cardiac biomarker for CHF diagnosis and a promising prognostic biomarker for CHF, which is consistent with previous reports. Nevertheless, the relationship between plasma level of cardiotrophin‐1 and pro‐BNP in CHF patients with various causes is unclear. Further large‐scale prospective studies are necessary to definitively validate this approach and to evaluate the potential prognostic implications of CT‐1 levels in CHF patients. In the future, we will perform a multi‐center study of more CHF patients and adopt multi‐biomarker strategy to more accurately diagnose patients with chronic heart failure.

## 4 CONCLUSION

In conclusion, we constructed a double‐antibody “sandwich”‐based chemiluminescent immunoassay for rapidly determine plasma level of CT‐1. Compared to the current reported methods for CT‐1 level analysis, the MPs‐CLIA assay greatly reduces the detection time by as short as 1 hour, whereas the widely applied enzyme‐linked immunosorbent assay (ELISA) requires more times of 4‐5 hours.^31^, ^32^, ^33^ And the assay is highly automated, avoiding the requirement of complicated operating procedures. Furthermore, systematic validation analysis revealed that the novel method has sufficient precision, accuracy, and reliability for quantitative CT‐1. This developed chemiluminescence immunoassay will be a powerful tool to further explore more clinical value of CT‐1.

## AUTHOR CONTRIBUTIONS

YP involved in protocol development, performed laboratory measurements, statistical analysis, and manuscript writing. XW involved in statistical analysis. YD and HW performed laboratory measurements. WL and PY collected clinical samples and data. ZT involved in protocol development and revised manuscript. All authors read and approved the final manuscript.

## ETHICS APPROVAL AND CONSENT TO PARTICIPATE

This study was approved by the Ethics Committee of the Second Affiliated Hospital, Zhejiang University School of Medicine. Written informed consent was obtained from all study participants before commencement of the study.
